# Supplementary material for: Heat adaptation of phage T7 under an extended genetic code
Source: Virus Evol. 2021 Dec 1;7(2):veab100. doi: 10.1093/ve/veab100 (PMC8923235; doi:10.1093/ve/veab100)
Supplement: veab100_Supp [file veab100_supp.zip › Sequencing_Analysis.zip!/Sequencing_Analysis/S2/evidence/SNP_5.html]

BRESEQ :: Evidence


| Predicted mutation | | | | | | |
| --- | --- | --- | --- | --- | --- | --- |
| evidence | seq id | position | mutation | annotation | gene | description |
| RA | NC\_001604.1 | 20,544 | G→A | intergenic (–/–) | – / – | –/– |

| Read alignment evidence... | | | | | | | | | | | |
| --- | --- | --- | --- | --- | --- | --- | --- | --- | --- | --- | --- |
|  | seq id | position | | ref | new | freq | score (cons/poly) | reads | annotation | genes | product |
| \* | NC\_001604.1 | 20,544 | 0 | G | A | 99.6% | 13808.6 / NA | 5399 | intergenic (–/–) | *–/–* | –/– |
| Reads supporting (aligned to +/- strand):  **ref** base G (5/6);  **major** base A (2648/2706);  **minor** base C (18/5);  **total** (2679/2720) | | | | | | | | | | | |
| Fisher's exact test for biased strand distribution *p*-value = 6.11e-03 | | | | | | | | | | | |
| Kolmogorov-Smirnov test that lower quality scores support variant *p*-value = 1.00e+00 | | | | | | | | | | | |

Only 100 of 5399 total aligned reads displayed.  

`GAGAAACGAACAGGACTTGCGGAGGATGGCGCAAAGTCTGTCTATGAGCGTTTAAAGAACGACCGTGCTCCCTATGAGACACGCGCTCAGAATTGCGCTCAATATACCATCCCATCATTGTTCCCTAAGGACTCCGATAACGCCTCTACAGATTATCAAACTCCGTGGCAAGCCGTGGGCGCTCGTGGTCTGAACAATCTAGCCTCTAAGCTCATGCTGGCTCTATTCCCTATGCAGACTTGGATGCGACTTACTATATCTGAATATGAAGCAAAGCAGTTACTGAGCGACCCCGATGGACTCGCTAAGGTCGATGAGGGCCTCTCGATGGTAGAGCGTATCATCATGAACTACATTGAGTCTAACAGTTACCGCGTGACTCTCTTTGAGGCTCTCAAACAGTTAGTCGTAGCTGGTAACGTCCTGCTGTACCTACCGGAACCGGAAGGGTCAAACTATAATCCCATGAAGCTGTACCGATTGTCTTCTTATGTGGTCCAACGAGACGCATTCGGCAACGTTCTGCAAATGGTGACTCGTGACCAGATAGCTTTTGGTGCTCTCCCTGAGGACATCCGTAAGGCTGTAGAAGGTCA  >  NC_001604.1/20246‑20841`  

                                                                                                                                                                                                                                                                                                          `|`                                                                                                                                                                                                                                                                                                           
    `aaCGAACAGGACTTGCGGAGGATGGCGCAAAGTCTGTCTATGAGCGTTTAAAGAACGACCGTGCTCCCTATGAGACACGCGCTCAGAATTGCGCTCAATATACCATCCCATCATTGTTCCCTAAGGACGCCGATAACGCCTCTACAGATTATCAAACTCCGTGGCAAGCCGTGGGCGCTCGTGGTCTGAACAATCTAGCCTCTAAGCTCATGCTGGCTCTATTCCCTATGCAGACTTGGATGCGACTTACTATATCTGAATATGAAGCAAAGCAGTTACTGAGCGACCCCGATGAACTCGc                                                                                                                                                                                                                                                                                                     <  1:220514/301‑1 (MQ=255)`  
      `cGAACAGGACTTGCGGAGGATGGCGCAAAGTCTGTCTATGAGCGTTTAAAGAACGACCGTGCTCCCTATGAGACACGCGCTCAGAATTGCGCTCAATATACCATCCCATCATTGTTCCCTAAGGACTCCAATAAAGCCTCTACAGATTATCAAACTCCGTGGCAAGCCGTGGGCGCTCGTGGTCTGAACAATCTAGCCTCTAAGCTCATGCTGGCTCTATTCCCTATGCAGACTTGGATGCGACTTACTATATCTGAATATGAAGCAAAGCAGTTACTGAGCGACCCCGATGAACTCGCTa                                                                                                                                                                                                                                                                                                   >  1:676514/1‑301 (MQ=255)`  
          `gtgggctcggagatgtgtataagagacaggtctATGAGCGTTTAAAGAACGACCGTGCTCCCTATGAGACACGCGCTCAGAATTGCGCTCAATATACCATCCCATCATTGTTCCCTAAGGACTCCGATAACGCCTCTACAGATTATCAAACTCCGTGGCAAGCCGTGGGCGCTCGTGGTCTGAACAATCTAGCCTCTAAGCTCATGCTGGCTCTATTCCCTATGCAGACTTGGATGCGACTTACTATATCTGAATATGAAGCAAAGCAGTTACTGAGCGACCCCGATGAACTCGCTAAGGt                                                                                                                                                                                                                                                                                               <  1:398797/272‑1 (MQ=255)`  
             `gACTTGCGGAGGATGGCGCAAAGTCTGTCTATGAGCGTTTAAAGAACGACCGTGCTCCCTATGAGACACGCGCTCAGAATTGCGCTCAATATACCATCCCATCATTGTTCCCTAAGGACTCCGATAACGCCTCTACAGATTATCAAACTCCGTGGCAAGCCGTGGGCGCTCGTGGTCTGAACAATCTAGCCTCTAAGCTCATGCTGGCTCTATTCCCTATGCAGACTTGGATGCGACTTACTATATCTGAATATGAAGCAAAGCAGTTACTGAGCGACCCCGATGAACTCGCTAAGGTCGa                                                                                                                                                                                                                                                                                            >  1:161395/1‑301 (MQ=255)`  
                       `ggaTGGCGCAAAGTCTGTCTATGAGCGTTTAAAGAACGACCGTGCTCCCTATGAGACACGCGCTCAGAATTGCGCTCAATATACCATCCCATCATTGTTCCCTAAGGACTCCGATAACGCCTCTACAGATTATCAAACTCCGTGGCAAGCCGTGGGCGCTCGTGGTCTGAACAATCTAGCCTCTAAGCTCATGCTGGCTCTATTCCCTATGCAGACTTGGATGCGACTTACTATATCTGAATATGAAGCAAAGCAGTTACTGAGCTACCCCGATGAACTCGCTAAGGTCGATgctgtctct                                                                                                                                                                                                                                                                                  >  1:182786/1‑293 (MQ=255)`  
                        `gaTGGCGCAAAGTCTGTCTATGAGCGTTTAAAGAACGACCGTGCTCCCTATGAGACACGCGCTCAGAATTGCGCTCAATATACCATCCCATCATTGTTCCCTAAGGACTCCGATAACGCCTCTACAGATTATCAAACTCCGTGGCAAGCCGTGGGCGCTCGTGGTCTGAACAATCTAGCCTCTAAGCTCATGCTGGCTCTATTCCCTATGCAGACTTGGATGCGACTTACTATATCTGAATATGAAGCAAAGCAGTTACTGAGCGACCCAGATGAACTCGCTAAGGTctgtctcttataca                                                                                                                                                                                                                                                                                 >  1:585582/1‑288 (MQ=255)`  
                          `tGGCGCAAAGTCTGTCTATGAGCGTTTAAAGAACGACAGTGCTCCCTATGAGACACGCGCTCAGAATTGCGCTCAATATACCATCCCATCATTGTTCCCTAAGGACTCCGATAACGCCTCTACAGATTATCAAACTCCGTGGCAAGCCGTGGGCGCTCGTGGTCTGAACAATCTAGCCTCTAAGCTCATGCTGGCTCTATTCCCTATGCAGACTTGGATGCGACTTACTATATCTGAATATGAAGCAAAGCAGTTACTGAGCGACCCCGATGAACTCGCTAAGGTCGATGAGGGCCTCTCg                                                                                                                                                                                                                                                                               <  1:168296/301‑1 (MQ=255)`  
                            `gcgcAAAGTCTTTCTATGAGCGTTTAAAGAACGACCGTGCTCCCTATGAGACACGAGCTCAGAATTGCGCTCAATATACCATCCCATCATTGTTCCCTAAGGACTCCGATAACGCCTCTACATATTATCAAACTCCGTGGCAAGCCGTGGGCGCTCGTGGTCTGAACAATCTAGCCTCTAAGCTCATGCTGGCTCTATTACATATGCAGACTTGGATGCGACTTACTATATCTGAATATGAAGCAAAGCAGTTACTGAGCGACCCCGATGAACTCGCTAAGGTCGATGAGGGCCTCTCGAt                                                                                                                                                                                                                                                                             <  1:810079/301‑1 (MQ=255)`  
                            `gcgcAAAGTCTGTCTATGAGCGTTTAAAGAACGACCGTGCTCCCTATGAGACACGCGCTCAGAATTGCGCTCAATATACCATCCCATCATTGTTCCCTAAGGACTCCGATAACGCCTCTACAGATTATCAAACTCCGTGGCAAGCCGTGGGCGCTCGTGGTCTGAACAATCTAGCCTCTAAGCTCATGCTGGCTCTATTCCCTATGCAGACTTGGATGCGACTTACTATATCTGAATATGAAGCAAAGCAGTTACTGAGCGACCCCGATGAACTCGCTAAGGTCGATGAGGGCCTCTCGAt                                                                                                                                                                                                                                                                             <  1:464451/301‑1 (MQ=255)`  
                               `aaaaGTCTGTCTATGAGCGTTTAAAGAACGACCGTGCTCCCTATGAGACACGCGCTCAGAATTGCGCTCAATATACCATCCCATCATTGTTCCCTAAGGACTCCGATAACGCCTCTACAGATTATCAAACTCCGTGGCAAGCCGTGGGCGCTCGTGGTCTGAACAATCTAGCCTCTAAGCTCATGCTGGCTCTATTCCCTATGCAGACTTGGATGCGACTTACTATATCTGAATATGAAGCAAAGCAGTTACTGAGCGACCCCGATGAACTCGCTAAGGTCGATGAGGGCCTCTCGATGGt                                                                                                                                                                                                                                                                          <  1:442459/300‑1 (MQ=255)`  
                                 `aggtctgtctATGAGCGTTTAAAGAACGACCGTGCTCCCTATGAGACACGCGCTCAGAATTGCGCTCAATATACCATCCCATCATTGTTCCCTAAGGACTCCGATAACGCCTCTACAGATTATCAAACTCCGTGGCAAGCCGTGGGCGCTCGTGGTCTGAACAATCTAGCCTCTAAGCTCATGCTGGCTCTATTCCCTATGCAGACTTGGATGCGACTTACTATATCTGAATATGAAGCAAAGCAGTTACTGAGCGACCCCGATGAACTCGCTAAGGTCGATGAGGGCCTCTCGATGGTag                                                                                                                                                                                                                                                                        <  1:236574/299‑1 (MQ=255)`  
                                   `gtctgtctATGAGCGTTTAAAGAACGACCGTGCTCCCTATGAGACACGCGCTCAGAATTGCGCTCAATATACCATCCCATCATTGTTCCCTAAGGACTCCGATAACGCCTCTACAGATTATCAAACTCCGTGGCAAGCCGTGGGCGCTCGTGGTCTGAACAATCTAGCCTCTAAGCTCATGCTGGCTCTATTCCCTATGCAGACTTGGATGCGACTTACTATATCTGAATATGAAGCAAAGCAGTTACTGAGCGACCCCGATGAACTCTCTAAGGTCGATGAGGGCCTCTCGATGGTagag                                                                                                                                                                                                                                                                      <  1:636056/301‑1 (MQ=255)`  
                                         `ttATGAGCGTTTAAAGACCGACCGTGCTCCCTATGAGACACGCGCTCAGAATTGCGCTCAATATACCATCCCATCATCGTTCCCTAAGGACTCCGATAACGCCTCTACAGATTATCAAACTCCGTGGCAAGCCGTGGGCGCTCGTGGTCTGAACAATCTAGCCTCTAAGCTCATGCTGGCTCTATTCCCTATGCAGACTTGGATGCGACTTACTATATCTGAATATGAAGCAAAGCAGTTACTGAGCGACCCCGATGAACTCGCTAAGGTCGATGAGGGCCTCTCGATGGTAGAGCGTatc                                                                                                                                                                                                                                                                <  1:735249/300‑1 (MQ=255)`  
                                          `tATGAGCGTTTAAAGAACGACCGTGCTCCCTATGAGACACGCGCTCAGAATTGCGCTCAATATACCATCCCATCATTGTTCCCTAAGGACTCCGATAACGCCTCTACAGATTATCAAACTCCGTGGCAAGCCGTGGGCGCTCGTGGTCTGAACAATCTAGCCTCTAAGCTCATGCTGGCTCTATTCCCTATGCAGACTTGGATGCGACTTACTATATCTGAATATGAAGCAAAGCAGTTACTGAGCGACCCCGATGAACTCGCTAAGGTCGATGAGGGCCTCTCGATGGTAGAGCGTatca                                                                                                                                                                                                                                                               <  1:479745/301‑1 (MQ=255)`  
                                           `aTGAGCGTTTAAAGAACGACCGTGCTCCCTATGAGACACGCGCTCAGAATTGCGCTCAATATACCATCCCATCATTGTTCCCTAAGGACTCCGATAACGCCTCTACAGATTATCAAACTCCGTGGCAAGCCGTGGGCGCTCGTGGTCTGAACAATCTAGCCTCTAAGCTCATGCTGGCTCTATTCCCTATGCAGACTTGGATGCGACTTACTATATCTGAATATGAAGCAAAGCAGTTACTGAGCGACCCCGATGAACTCGCTAAGGTCGATGAGGGCCTCTCGATGGTAGAGCGTatcat                                                                                                                                                                                                                                                              <  1:824126/301‑1 (MQ=255)`  
                                                 `atttAAAGAACGACCATGCTCCCTATGAGAAACGCGCTCAGAATTGCGCTCAATATACCATCCCATCATTGTTCCCTAAGGACTCCGATAACGCCTCTACAGATTATCAAACTCCGTGGCAAGCCGTTGGCGCTCGTGGTCTGAACAATCTAGCCTCTAAGCTCATGCT‑GCTCTATTCCCTATGCAGACTTGGATGCGACTTACTATATCTGAATATGAAGCAAAGCAGTTACTGAGCGACCCCGATGAACTCGCTAAGGTCGATGAGGGCCTCTCGATGGTAGAGCGTATCATCATGagc                                                                                                                                                                                                                                                       <  1:762911/300‑3 (MQ=255)`  
                                                  `tttAAAGAACGACCGTGCTCCCTATGAGACACGCGCTCAGAATTGCGCTCAATATACCATCCCATCATTGTTCCCTAAGGACTCCGATAACGCCTCTACAGATTATCAAACTCCGTGGCAAGCCGTGGGCGCTCGTGGTCTGAACAATCTAGCCTCTAAGCTCATGCTGGCTCTATTCCCTATGCAGACTTGGATGCGACTTACTATATCTGAATATGAAGCAAAGCAGTTACTGAGCGACCCCGATGAACTCGCTAAGGTCGATGAGGGCCTCTCGATGGTAGAGCGTATCATCATGAAc                                                                                                                                                                                                                                                       <  1:213499/301‑1 (MQ=255)`  
                                                  `tttAAAGAACGACCGTGCTCACTATGAGACACGCGCTCAGAATTGCGCTCAATATACCATCCCATCATTGTTCCCTAAGGACTCCGATAACGCCTCTACAGATTATCAAACTCCGTGGCAAGCCGTGGGCGCTCGTGGTCTGAACAATCTAGCCTCTAAGCTCATGCTGGCTCTATTCCCTATGCAGACTTGGATGCGACTTACTATATCTGAATATGAAGCAAAGCAGTTACTGAGCGACCCCGATGAACTCGCTAAGGTCGATGAGGGCCTCTCGATGGTAGAGCGTATCATCATGAAc                                                                                                                                                                                                                                                       <  1:488038/301‑1 (MQ=255)`  
                                                     `aaaGAACGACCGTGCTCCCTATGAGACACGCGCTCAGAATTGCGCTCAATATACCATCCCATCATTGTTCCCTAAGGACTCCGATAACGCCTCTACAGATTATCAAACTCCGTGGCAAGCCGTGGGCGCTCGTGGTCTGAACAATCTAGCCTCTAAGCTCATGCTGGCTCTATTCCCTATGCAGACTTGGATGCGACTTACTATATCTGAATATGAAGCAAAGCAGTTACTGAGCGACCCCGATGAACTCGCTAAGGTCGATGAGGGCCTCTCGATGGTAGAGCGTATCATCATGAACTAc                                                                                                                                                                                                                                                    >  1:338653/1‑301 (MQ=255)`  
                                                       `tgtataagagacagcccTATGAGACACGCGCTCAGAATTGCGCTCAATATACCATCCCATCATTGATCCCTAAGGACTCCGATAACGCCTCTACAGATTATCAAACTCCGTGGCAAGCCGTGGGCGCTCGTGGTCTGAACAATCTAGCCTCTAAGCTCATGCTGGCTCTATTCCCTATGCAGACTTGGATGCGACTTACTATATCTGAATATGAAGCAAAGCAGTTACTGAGCGACCCCGATGAACTCGCTAAGGTCGATGAGGGCCTCTCGATGGTAGAGCGTATCATCATGAACTACAt                                                                                                                                                                                                                                                  <  1:685715/287‑1 (MQ=255)`  
                                                       `agagacagcGTGCTCCCTATGAGACAAGCGCTCAGAATTGCGCTCAAGATACCATCCCATCATTGTGCCCTAAGGACTCCGATAACGCCTCTACAGATTATCAAACTCCGTGGCAAGCCGTGGGCGCTCGTGGTCTGAACAATCTAGCCGCTAAGCTCATGCTGGCTCTATTCCCTATGCAGACTTGGATGCGACTTACTATATCTGAATATGAAGCAAAGCAGTTACTGAGCGACCCCGATGAACTCGCTAAGGTCGATGAGGGCCTCTCGATGGTAGAGCGTATCATCATGAACTACAt                                                                                                                                                                                                                                                  <  1:660428/293‑1 (MQ=255)`  
                                                       `aGAACGACCGTGCTCCCTATGAGACACGCGCTCAGAATTGCGCTCAATATACCATCCCATCATTGTTCCCTAAGGACTCCGATAACGACTCTACAGATTATCAAACTCCGTGGCAAGCCGTGGGCGCTCGTGGTCTGAACAATCTAGCCTCTAAGCTCATGCTGGCTCTATTCCCTATGCAGACTTGGATGCGACTTACTATATCTGAATATGAAGCAAAGCAGTTACTGAGCGACCCCGATGAACTCGCTAAGGTCGATGAGGGCCTCTCGATGGTAGAGCGTATCATCATGAACTACAt                                                                                                                                                                                                                                                  <  1:256712/301‑1 (MQ=255)`  
                                                            `gACCGTGCTCCCTATGAGACACGCGCTCAGAATTGCGCTCAATATACCATCCCATCATTGTTCCCTAAGGACTCCGATAACGCCTCTACAGATTATCAAACTCCGTGGCAAGCCGTGGGCGCTCGTGGTCTGAACAATCTAGCCTCTAAGCTCATGCTGGCTCTATTCCCTATGCAGACTTGGATGCGACTTACTATATCTGAATATGAAGCAAAGCAGTTACTGAGCGACCCCGATGAACTCGCTAAGGTCGATGAGGGCCTCTCGATGGTAGAGCGTATCATCATGAACTACATTGAGt                                                                                                                                                                                                                                             <  1:428695/301‑1 (MQ=255)`  
                                                            `gACCGTGCCCCCTATGAGACACGCGCTCAGAATTGCGCTCAAAATACCATCCCAGCATTTTTCCCTAAGGACTCCGATAACGCCTCTACAGATTATCAAACTCCGTGGCAAGCCGTGGGCGCTAGTGGTCTGAACAATCTAGCCTCTAAGCTCATGCTGGCTCTATTCCCTAGGCAGACTTGGATGCGACTTACTATATCTGAATATGAAGCAAAGCAGTTACTGAGCGACCCCGATGAACTCGCTAAGGTCGATGAGGGCCTCTCGATGGTAGAGCGTATCATCATGAACTACATTGAGt                                                                                                                                                                                                                                             <  1:294458/301‑1 (MQ=255)`  
                                                                 `tGCTCCCTATGAGACACGCGCTCAGAATTGCGCTCAATATACCATCCCATCATTGTTCCCTAAGGACTCCGATAACGCCTCTACAGATTATAAAACTCCGTGGCAAGCCGTGGGCGCTCGTGGTCTGAACAATCTAGCCTCTAAGCTCATGCTGGCTCTATTCCCTATGCAGACTTGGATGCGACTTACTATATCTGAATATGAAGCAAAGCAGTTACTGAGCGACCCCGATGAACTCGCTAAGGTCGATGAGGGCCTCTCGATGGTAGAGCGTATCATCATGAACTACATTGAGTCTAAc                                                                                                                                                                                                                                        <  1:100217/301‑1 (MQ=255)`  
                                                                     `cccTATGAGACACGCGCTCAGAATTGCGCTCAATATACCATCCCATCATTGTTCCCTAAGGACTCCGATAACGCCTCTACAGATTATCAAACTCCGTGGCAAGCCGTGGGCGCTCGTGGTCTGAACAATCTAGCCTCTAAGCTCATGCTGGCTCTATTCCCTATGCAGACTTGGATGCGACTTACTATATCTGAATATGAAGCAAAGCAGTTACTGAGCGACCCCGATGAACTAGCTAAGGTCGATGAGGGCCTATCGATGGTAGAGCGGATCATCATGactgacccgtaaacacaactcc                                                                                                                                                                                                                                    >  1:286289/1‑280 (MQ=255)`  
                                                                     `cccTATGAGACACGCGCTCAGAATTGCGCTCAATATACCATCCCATCATTGTTCCCTAAGGACTCCGATAACGCCTCTACAGATTATCAAACTCCGAGGCAAGCCGTGGGCGCTCGTGGTCTGAACAATCTAGCCTCTAAGCTCATGCTGGCTCTATTCCCTATGCAGACTTGGATGCGACTTACTATATCTGAATATGAAGCAAAGCGGTTACTGAGCGACCCCGATGAACTCGCTAAGGTCGATGAGGGCCCCTCGATGGTAGAGCGTATCATCATGAACTACATTGAGTCTAACAGtt                                                                                                                                                                                                                                    >  1:206962/1‑301 (MQ=255)`  
                                                                      `agatgtgtataagagacaggAATTGCGCTCAATATACCATCCCATCATTGGTCCCTAAGGACTCCGATAACGCCTCTACAGATTATCAAACTCCGTGGCAAGCCGTGGGCGCTCGTGGTCTGAACAATCTAGCCTCTAAGCTCATGCTGGCTCTATTCCCTAGGCAGACTTGGATGCGACTTACTATATCTGAATATGAAGCAAAGCAGTTACTGAGCGACCCCGATGAACTCGCTAAGGTCGATTAGGGCCTCTCGATGGTAGAGCGTATCATCATGAACTACATTGAGTCTAACAgctg                                                                                                                                                                                                                                   <  1:196996/282‑4 (MQ=255)`  
                                                                       `ggggctcggagatgtgtataagagacagcAATATACCATCCCATCATTGTTCCCTAAGGACTCCGATAACGCCTCTACAGATTATCAAACTCCGTGGCAAGCCGTGGGCGCTCGTGGTCTGAACAATCTAGCCTCTAAGCTCATGCTGGCTCTATTCCCTATGCAGACTTGGATGCGACTTACTATATCTGAATATGAAGCAAAGCAGTTACTGAGCGACCCCGATGAACTCGCTAAGGTCGATGAGGGCCTCTCGATGGTAGAGCGTATCATCATGAACTACATTGAGTCTAACAGTTAc                                                                                                                                                                                                                                  <  1:384112/273‑1 (MQ=255)`  
                                                                       `cTATGAGACACGCGCTCAGAATTGCGCTCAATATACCATCCCATCATTGTTCCCTAAGGACTCCGATAACGCCTCTACAGATTATCAAACTCCGTGGCAAGCCGTGGGCGCTCGTGGTCTGAACAATCTAGCCTCTAAGCTCATGCTGGCTCTATTCCCTATGCAGACTTGGATGCGACTTACTATATCAGAATATGAAGCAAAGCAGTTACTGAGCGACCCCGATGAACTCGCTAAGGTCGATGAGGGCCTCTCGATGGTAGAGCGTATCATCATGAACTACATTGAGTCTAACAGTTAc                                                                                                                                                                                                                                  >  1:715297/1‑301 (MQ=255)`  
                                                                                         `gAATTGCGCTCAATATACCATCCCATCATTTTTCCCTAAGGACTCCGATAACGCCTCTACAGATTATCAAACTCCGTGGCAAGCCGTGGGCGCTCGTGGTCTGAACAATCTAGCCTCTAAGCTCATGCTGGCTCTATTCCCTATGCAGACTTGGATGCGACTTACTCTATCTGAATAAGAAGCAAAGCAGTTACTGAGCGACCCCGATGAACTCGCTAAGGTCGATCAGTGCCTCTCGATGGTAGAGCGTATCATCATGAACTACATTGAGTCTAACAGTTACCGCGTGACTCCCTTTGAg                                                                                                                                                                                                                >  1:817529/1‑301 (MQ=255)`  
                                                                                         `gAATTGCGCTCAATATACCATCCCATCATTGTTCCCTAAGGACTCCGATAACGCCTCTACAGATTATCAAACTCCGTGGCAAGCCGTGGGCGCTCGTGGTCTTAACAATCTAGCCTCTAAGCTCATGCTGGCTCTATTCCCTATGCAGACTTGGATGCGACTTACTATATCTGAATATGAAGCAAAGCAGTTACTGAGCGACCCCGATGAACTCGCTAAGGTCGATGAGGGCCTCTCGATGGTAGAGCGTATCATCATGAACTACATTGAGTCTAACAGTTACCGCGTGACTCTCTTTGAg                                                                                                                                                                                                                >  1:10807/1‑301 (MQ=255)`  
                                                                                           `tataagagacagataCCATCCCATCATTGTTCCCTAATGTCTCCGATAACTCCTCTACAGATTATCAAACTCCGTGGCAAGCCGTGGGCGCTCGTGGTCTGAACAATCTAGCCTCTAAGCTCATGCTGGCTCTATTCCCTATGCAGACTTGGATGCGACTTACTATATCTGAATATGAAGCAAAGCAGTTACTGAGCGACCCCGATGAACTCGCTAAGGTCGATGAGGGCCTCTCGATGGTAGAGCGTATCATCATGAACTACATTGAGTCTAACAGTTACCGCGTGACTCTCTTTGAGGc                                                                                                                                                                                                              <  1:786913/289‑1 (MQ=255)`  
                                                                                           `gtataagagacagtaCCATCCCATCATTGTTCCCTAAGGACTCCGATAACGCCTCTACAGATTATCAAACTCCGTGGCAAGCCGTGGGCGCTCGTGGTCTGAACAATCTAGCCTCTAAGCTCATGCTGGCTCTATTCCCTATGCAGACTTGGATGCGACTTACTATATCTGAATATGAAGCAAAGCAGTTACTGAGCGACCCCGATGAACTCGCTAAGGTCGATGAGGGCCTCTCGATGGTAGAGCGTATCATCATGAACTACATTGAGTCTAACAGTTACCGCGTGACTCTATTTGAGGc                                                                                                                                                                                                              <  1:277969/288‑1 (MQ=255)`  
                                                                                                   `cAATATACCATCCCATCATTGTTCCCTAAGGACTCCGATAACGCCTCTACAGATTATCAAACTCCGTGGCAAGCCGTGGGCGCTCGTGGTCTGAACAATCTAGCCTCTAAGCTCATGCTGGCTCTATTCCCTATGCAGACTTGGATGCGACTTACTATATCTGAATATGAAGCAAAGCAGTTACTGAGCGACCCCGCTGAACTCGCTAAGGTCGATGAGGGCTTCTCGATGGTAGAGCGTATAATCATGAACTACATTTAGTCTAACAGTTACCGCGTGACTCTCTTTGAGGCTCTCAAAc                                                                                                                                                                                                      >  1:794866/1‑301 (MQ=255)`  
                                                                                                   `cAATATACCATCCCATCATTGTTCCCTAAGGACTCCGATAACGCCTCTACAGATTATCAAACTCCGTGGCAAGCCGTGGGCGCTCGTGGTCTGAACAATCTAGCCTCTAAGCTCATGCTGGCTCTATTCCCTATGCAGACTTGGATGCGACTTACTATATCTGAATATGAAGCAAAGCAGTTACTGAGCGACCCCGATGAACTCGCTAAGGTCGATGAGGGCCTCTCGATGGTAGAGCGTATCATCATGAACTACATTGAGTCTAACAGTTACCGCGTGACTCTCTTTGAGGCTCTCaaaa                                                                                                                                                                                                      >  1:301367/1‑300 (MQ=255)`  
                                                                                                   `cAATATACCATCCCATCATTGTTCCCTAAGGACTCCGATAACGCCTCTACAGATTATCAAACTCCGTGGCAAGCCGTGGGCGCTCGTGGTCTGAACAATCTAGCCTCTAAGCTCATGCTGGCTCTATTCCCTATGCAGACTTGGATGCGACTTACTATATCTGAATATGAAGCAAAGCAGTTACTGAGCGACCCCGATGAACTCGCTAAGGTCGATGAGGGCCTCTCGATGGTAGAGCGTATCATCATGAACTACATTGAGTCTAACAGTTACCGCGTGACTCTCTTTGAGGCTCTCAAAc                                                                                                                                                                                                      >  1:123464/1‑301 (MQ=255)`  
                                                                                                   `cAATATACCATCCCATCATTGTTCCCTAAGGACTCCGATAACGCCTCTACAGATTATCAAACTCCGTGGCAAGCCGTGGGCGCTCGTGGTCTGAACAATCTAGCCTCTAAGCTCATGCTGGCTCTATTCCCTATGCAGACTTGGATGCGACTTACTATATCTGAATATGAAGCAAAGCAGTTACTGAGCGACCCCGATGAACTCGCTAAGGTCGATGAGGGCCTCTCGATGGTAGAGCGTATCATCATGAACTACATTGAGTCTAACAGTTACCGCGTGACTCTCTTTGAGGCTATCAAAc                                                                                                                                                                                                      >  1:643341/1‑301 (MQ=255)`  
                                                                                                   `cAATATACCATCCCATCATTGTTCCCTAAGGACTCCGATAACGCCTCTACAGATTATCAAACTCCGTGGCAAGCCGTGGGCGCTCGTGGTCTGAACAATCTAGCCTCTAAGCTCATGCTGGCTCTATTCCCTATGCAGACTTGGATGCGACTTACTATATCTGAATATGAAGCAAAGCAGTTACTGAGCGACCCCGATGAACTCGCTAAGGTCGATGAGGGCCTCTCGATGGTAGAGCGTAACATCATGAACTACATTGAGTCTAAAAGTTACCGCGTGACTCTCTTTGAGGCCCTCaaaa                                                                                                                                                                                                      >  1:367260/1‑300 (MQ=255)`  
                                                                                                   `cAATATACCATCCCATCATTGTTCCCTAAGGACTCCGATAACGCCTCTACAGATTATCAAACTCCGTGGCAAGCCGTGGGCGCTCGTGGTCTGAACAATCTAGCCTCTAAGCTCATGCTGGCTCTATTCCCTATGCAAACTTGGATGCGACTTACTATATCTGAATATGAAGCAAAGCAGTTACTGAGCGACCCCGATGAACTCGCTAAGGTCGATGAGGGCCTCTCGATGGTAGAGCGTATCATCATGAACTACATTGAGTCTAACAGTTACCGCGTGACTCTCTTTGAGGCTCTCaaaa                                                                                                                                                                                                      >  1:331880/1‑300 (MQ=255)`  
                                                                                                   `aaaTATACCATCCCATCATTGTTCCCTAAGGAATACGATAACGCATCTACAGATTATCAAACTCCGTGGCAAGCCGTGGGCGCTCGTGGTCTGAACAATCTAGCCTCTAAGCTCATGCTGGATCTCTTCCCTATGCAGACTTGGATGCGCCTCACTATATCGGAAAATGAAGCAAAGCAGTTACTGAGAGACCCCGATGAACTAGCTAAGGTCGATGAGGGCCTGTCGATGGTAGAGCGTATCAGCACGAACTACATGGAGTCTAACAGTTACCGCGGGAACCTCTTTGAGGCTTTCaaaa                                                                                                                                                                                                      >  1:458383/2‑300 (MQ=255)`  
                                                                                                     `atatACCATCCCATCATTGTTCCCTAAGGACTCCGATAACGCCTCTACAGATTATCAAACTCCGTGGCAAGCAGTGGGCGCTCGTGGTCTGAACAATCTAGCCTCTAAGCTCATGCTGGCTCTATTCCCTATGCAGACTTGGATGCGACTTACTATATCTGAATATGAAGCAAAGCAGTTACTGAGCGACCCCGATGAACTCGCTAAGGTCGATGAGGGCCTCTCGATGGTAGAGCGTATCATCATGAACTACATTGAGTCTAACAGTTACCGCGTGACTCTCTTTGAGGCTCTCAAACAg                                                                                                                                                                                                    <  1:84835/301‑1 (MQ=255)`  
                                                                                                       `ataCCATCCCATCATTGTTCCCTAAGGACTCCGATAACGCCTCTACAGATTATCAAACTCCGTGGCAATCCGTGGGCGCTCGTGGTCTGAACAATCTAGCCTCTAAGCTCATGCTGGCTCTATTCCCTATGCAGACTTGGATGCGACTTACTATATCTGAATATGAAGCAAAGCAGTTACTGAGCGACCCCGATGAACTCGCTAAGGTCGATGAGGGCCTCTCGATGGTAGAGCGTATCATCATGAACTACATTGAGTCTAACAGTTACCGCGTGACTCTCTTTGAGGCTCTCAAACAGtt                                                                                                                                                                                                  >  1:533190/1‑301 (MQ=255)`  
                                                                                                          `ccatcccatcATTGTTCCCTAAGGACTCCGATAACGCCTCTACAGATTATCAAACTCCGTGGCAAGCCGTGGGCGCTCGTGGTCTGAACAATCTAGCCTCTAAGCTCATGCTGGCTCTATTCCCTATGCAGACTTGGATGCGACTTACTATATCTGAATATGAAGCAAAGCAGTTACTGAGCGACCCCGATGAACTCGCTAAGGTCGATGAGGGCCTCTCGATGGTAGAGCGTATCATCATGAACTACATTGAGTCTAACAGTTACCGCGTGACTCTCTTTGAGGCTCTCAAACAGTTAGt                                                                                                                                                                                               <  1:115573/301‑1 (MQ=255)`  
                                                                                                          `ccatcccatcATTGTTCCCTAAGGACTCCGATAACGCCTCTACAGATTATCAAACTCCGTGGCAAGCCGTGGGCGCTCGTGGTCTGAACAATCTAGCCTCTAAGCTCATGCTGGCTCTATTCCCTATGCAGACTTGGATGCGACTTACTATATCTGAATATGAAGCAAAGCAGTTACTGAGCGACCCCGATGAACTCGCTAAGGTCGATGAGGGCCTCTCGATGGTAGAGCGTATCATCATGAACTACATTGAGTCTAACAGTTACCGCGTGACTCTCTTTGAGGCTCTCAAACAGTTAGt                                                                                                                                                                                               >  1:605764/1‑301 (MQ=255)`  
                                                                                                             `tcccatcATTGTTCCCTAAGGACTCCGATAACGCCTCTACAGATTATCAAACTCCGTGGCAAGCCGTGGGCGCTCGTGGTCTGAACAATCTAGCCTCTAAGCTCATGCTGGCTCTATTCCCTATGCAGACTTGGATGCGACTTACTATATCTGAATATGAAGCAAAGCAGTTACTGAGCGACCCCGATGAACTCGCTAAGGTCGATGAGGGCCTCTCGATGGTAGAGCGTATCATCATGAACTACATTGAGTCTAACAGTTACCGCGTGACTCTCTTTGAGGCTCTCAAACAGTTAGTCGt                                                                                                                                                                                            >  1:650590/1‑301 (MQ=255)`  
                                                                                                                `aagagacagtCCCTAAGGACTCCGATAACGCCTCTACAGATTATCAAACTCCGTGGCAAGCCGTGGGCGCTCGTGGTCTGAACAATCTAGCCTCTAAGCTCATGCTGGCTCTATTCCCTATGCAGACTTGGATGCGACTTACTATATCTGAATATGAAGCAAAGCAGTTACTGAGCGACCCCGATGAACTCGCTAAGGTCGATGAGGGCCTCTCGATGGTAGAGCGTATCATCATGAACTACATTGAGTCTAACAGTTACCGCGTGACTCTCTTTGAGGCTCTCAAACAGTTAGTCGGAGc                                                                                                                                                                                         <  1:502784/292‑1 (MQ=255)`  
                                                                                                                       `gTTCCCTAAGGACTCCGATAACGCCTCTACAGATTATCAAACTCCGTGGCAAGCCGTGGGCGCTCGTGGTCTGAACAATCTAGCCTCTAAGCTCATGCTGGCTCTATTCCCTATGCAGACTTGGATGCGACTTACTATATCTGAATATGAAGCAAAGCAGTTACTTAGCGACCCAGATGAACTCGCTAAGGTCGATGAGGGCCTCTCGATGGTAGAGCGTATCATCATGAACTACCTTTAGTCTAACAGTTACCGCGTGACTATCTTTGAGGCTCTCCAACAGTTAGTCGTAGCTGGTAAc                                                                                                                                                                                  >  1:449851/1‑301 (MQ=255)`  
                                                                                                                                `ggACTCCGATAACGCCTCTACAGATTATCAAACTCCGTGGCAAGCCGTGGGCGCTCGTGGTCTGAACAATCTAGCCTCTAAGCTCATGCTGGCTCTATTCCCTATGCAGACTTGGATGCGACTTACTATATCTGAATATGAAGCAAAGCAGTTACTGAGCGACCCCGATGAACTCGCTAAGGTCGATGAGGGCCTCTCGATGGTAGAGCGTATCATCATGAACTACATTGAGTCTAACAGTTACCTCGTGACTCTCTTTGAGGCTCTCAAACAGTTAGTCGTAGCTGGTAACGTCctgctg                                                                                                                                                                         >  1:667645/1‑301 (MQ=255)`  
                                                                                                                                   `gcggagatgggtataagagacagaTCAAACTCCGTGGCAAACCGTGGGCGCTCGTGGTCTGAACAATCTAGCCACTAAGCTCATGCTGGCTCTATTCCCTATGCAGACTTGGATGCGACTTACTATATCTGAATATGAAGCAAAGCAGTTACTGAGCGACCCAGATGAACTCGCTAAGGTCGATGAGGGCCTCTCGATGGTAGAGCGTATCATCATGAACGACATTGAGTCTAACAGTTACCGCGTGACTCTCTTTGAGGCTCTCAAACAGTTAGTCGTAGCTGGTACCGTCCTGCTGtac                                                                                                                                                                      <  1:323424/278‑1 (MQ=255)`  
                                                                                                                                     `ccGATAACGCCTCTACAGATTATCAAACTCCGTGGCAAGCCGTGGGCGCTCGTGGTCTGAACAATCTAGCCTCTAAGCTCATGCTGGCTCTATTCCCTATGCAGACTTGGATGCGACTTACTATATCTGAATATGAAGCAAAGCAGTTACTGAGCGACCCCGATGAACTCGCTAAGGTCGATGAGGGCCTCTCGATGGTAGAGCGTATCATCATGAACTACATTGAGTCTAACAGTTACCGCGTGACTCTCTTTGAGGCTCTCAAACAGTTAGTCGTAGCTGGTAACGTCCTGCTGtacct                                                                                                                                                                    <  1:517296/301‑1 (MQ=255)`  
                                                                                                                                     `ccGATAACGCCTCTACAGATTATCAAACTCCGTGGCAAGCCGTGGGCGCTCGTGGTCTGAACAATCTAGCCTCTAAGCTCATGCTGGCTCTATTCCCTATGCAGACTTGGATGCGACTTACTATATCTGAATATGAAGCAAAGCAGTTACTGAGCGACCCCGATGAACTCGCTAAGGTCGATGAGGGCCTCTCGATGGTAGAGCGTATCATCATGAACTACATTGAGTCTAACAGTTACCGCGTGACTCTCTTTGAGGCTCTCAAACAGTTAGTCGTAGCTGGTAACGTCCTGCTGtacct                                                                                                                                                                    <  1:769725/301‑1 (MQ=255)`  
                                                                                                                                            `cGCCTCTACAGATTATCAAACTCCGTGGCAAGCCGTGGGCGCTCGTGGTCTGAACAATCTAGCCTCTAAGCTCATGCTGGCTCTATTCCCTATGCAGACTTGGATGCGACTTACTATATCTGAATATGAAGCAAAGCAGTTACTGAGCGACCCCGATGAACTCGCTAAGGTCGATGAGGGCCTCTCGATGGTAGAGCGTATCATCATGAACTACATTGAGTCTAACAGTTACCGCGTGACTCTCTTTGAGGCTCTCAAACAGTTAGTCGTAGCTGGTAACGTCCTGCTGTACCTaccggaa                                                                                                                                                             >  1:437034/1‑301 (MQ=255)`  
                                                                                                                                            `cGCCTCTACAGATTATCAAACTCCGTGGCAAGCCGTGGGCGCTCGTGGTCTGAACAATCTAGCCTCTAAGCTCATGCTGGCTCTATTCCCTATGCAGACTTGGATGCGACTTACTATATCTGAATATGAAGCAAAGCAGTTACTGAGCGACCCCGATGAACTCGCTAAGGTCGATGAGGGCCTCTCGATGGTAGAGCGTATCATCATGAACTACATTGAGTCTAACAGTTACCGCGTGACTCTCTTTGAGGCTCTCAAACAGTTAGTCGTAGCTGGTAACGTCCTGCTGTACCTacaggaa                                                                                                                                                             >  1:376403/1‑301 (MQ=255)`  
                                                                                                                                              `ccTCTACAGATTATCAAACTCCGTGGCAAGCCGTGGGCGCTCGTGGTCTGAACAATCTAGCCTCTAAGCTCATGCTGGCTCTATTCCCTATGCAGACTTGGATGCTACTTACTATATCTGAATATGAAGCAAAGCAGTTACTGAGCGACCCCGATGAACTCGCTAAGGTCGATGAGGGCCTCTCGATGGTAGAGCGTATCATCATGAACTACATTGAGTCTAACCGTTACCGCGTGACTCTCTTTGAGGCTCTCAAACAGTTAGTCGTAGCTTGTAACGTCCTGCTGTACCTaccggaacc                                                                                                                                                           >  1:742821/1‑301 (MQ=255)`  
                                                                                                                                                `tctACAGATTATCAAACTCCGTGGCAAGCCGTGGGCGCTCGTGGTCTGAACAATCTAGCCTCTAAGCTCATGCTGGCTCTATTCCCTATGCAGACTTGGATGCGACTTACTATATCTGAATATGAAGCAAAGCAGTTACTGAGCGACCCCGATGAACTCGCTAAGGTCGATGAGGGCCTCTCGATGGTAGAGCGTATCATCATGAACTACATTGAGTCTAACAGTTACCGCGTGACTCTCTTTGAGGCTCTCAAACAGTTAGTCGTAGCTGGTAACGTCCTGCTGTACCTaccggaaccgg                                                                                                                                                         >  1:14421/1‑301 (MQ=255)`  
                                                                                                                                                 `ctACAGATTATCAAACTCCGTGGCAAGCCGTGGGCGCTCGTGGTCTGAACAATCTAGCCTCTAAGCTCATGCTGGCTCTATTCCCTATGCAGACTTGGATGCGTCTTACTATATCTGAATATGAAGCAAAGCAGTTACTGAGCGACCCCGATGAACTCGCTAAGGTCGATGAGGGCCTCTCGATGGTAGAGCGTATCATCATGAACTACATTTAGTCTAACAGTTACCGCGTGACTCTCTTTGAGCCTCTCAAACAGTTAGTCGTAGCTGGTAACGTCCTGCTTTACCTaccggaaccgga                                                                                                                                                        >  1:548508/1‑301 (MQ=255)`  
                                                                                                                                                  `tACAGATTATCAAACTCCGTGGCAAGCCGTGGGCGCTCGTGGTCTGAACAATCTAGCCTCTAAGCTCCTGCTGGCTCTATTCCCTATGCAGACTTGGATGCGACTTACTATATCTGAATATGAAGCAAAGCAGTTACTGAGCGACCCCGATGAACTCGCTAAGGTCGATGAGGGCCTCTCGATGGTAGAGCGTATCATCATGAACTACATTGAGTCTAACAGTTACCGCGTGACTCTCTTTGAGGCTCTCAAACAGTTAGTCGTAGCTGGTAACGTCCTGCTGTACCTAccggaaccggaa                                                                                                                                                       <  1:153508/301‑1 (MQ=255)`  
                                                                                                                                                  `gtataagagacagaCTCCGTGGCAAGCCGTGGGCGCTCGTGGTCTGAACAATCTAGCCTCTAAGCTCATGCTGGCTCTATTCCCTATGCAGACTTGGATGCGACTTACTATATCTGAATATGAAGCAAAGCAGTTACTGAGCGACCCCGATGAACTCGCTAAGGTCGATGAGGGCCTCTCGATGGTAGAGCGTATCATCATGAACTACATTGAGTCTAACAGTTACCGCGTGACTCTCTTTGAGGCTCTCAAACAGTTAGTCGTAGCTGGTAACGTCCTGCTGTACCTAccggaaccggaa                                                                                                                                                       <  1:344834/288‑1 (MQ=255)`  
                                                                                                                                                    `cAGATTATCAAACTCCGTGGCAAGCCGTGGGCGCTCGTGGTCTGAACAATCTAGCCTCTAAGCTCATGCTGGCTCTATTCCCTATGCAGACTTGGATGCGACTTACTATATCTGAATATGAAGCAAAGCAGTTACTGAGCGACCCCGATGAACTCGCTAAGGTCGATGAGGGCCTCTCGATGGTAGAGCGTATCATCATGAACTACATTGCGTCTAACAGTTACCGCGTGACTCTCTTTGAGGCTCTCCAACAGTTAGTCGTAGCTGGTAACGTCCTGCTGTACCTAacggaaccggaacg                                                                                                                                                     >  1:244146/1‑299 (MQ=255)`  
                                                                                                                                                      `gATTATCAAACTCCGTGGCAAGCCGTGGGCGCTCGTGGTCTGAACAATCTAGCCTCTAAGCTCATGCTGGCTCTCTTCCCTATGCAGACTTGGATGCGACTTACTATATCTGAATATGAAGCAAAGCAGTTACTGAGCGACCCCGATGAACTCGCTAAGGTCGATGAGGGCCTCTCGATGGTAGAGCGTATCATCATGAACTACATTGAGTCTAACAGTTACCGCGTGACTCTCTTTGAGGCTCTCCAACAGTTAGTCGTAGCTGGTAATGTCCTGCTGTACCTACCGGAACCGGAAgggg                                                                                                                                                   >  1:358352/1‑300 (MQ=255)`  
                                                                                                                                                                           `gCCGTGGGCGCTCGGGGTCTGAAGAATCTAGCCTCTAAGCTCATGCTGGCTCTATTCCCTATGCAGACTTGGATGCGACTTACTATATCTGAATATGAAGCAAAGCAGTTACTGGGCGACCCCGATGAACTCGCTAAGGTCGATGAGGGCCTCTCGATGGTAGAGCGTATCATCATGAACTACATTGAGTCTAACAGTTACCGCGTGACTCTCTTTGAGGCTCTCAAACAGTTAGTCGTAGCTGGTAACGTCCTGCTGTACCTACCGGAACCGGAAGGGTCAAACTATAATCCCATGAAGc                                                                                                                              <  1:777291/301‑1 (MQ=255)`  
                                                                                                                                                                                `gagacagcGTGGTCTGAACAATCTAGCCTCTAAGCTCATGCTGGCTCTATTCCCTATGCAGACTTGGATGCGACTTACTATATCTGAATATGAAGCAAAGCAGTTACTGAGCGACCCCGATGAACTCGCTAAGGTCGATGAGGGCCTCTCGATGGTAGAGCGTATCATCATGAACTACATTGAGTCTAACAGTTACCGCGTGACTATCTTTGAGGCTCTCAAACAGTTAGTCGTAGCTGGTAACGTCCTACTGTACCTACCGGAACCGGAAGGGTCAAACTATAATCCCATGAAGCTGTAc                                                                                                                         <  1:70737/294‑1 (MQ=255)`  
                                                                                                                                                                                     `gacagggTCTGAACAATCTAGCCTCTAAGCTCATGCTGGCTCTATTCCCTATGCAGACTTGGATGCGACTTACTATATCTGAATATGAAGCAAAGCAGTTACTGAGCGACCCCGATGAACTCGCTAAGGTCGATGAGGGCCTCTCGATGGTAGAGCGTATCATCATGAACTACATTGAGTCTAACAGTTACCGCGTGACTCTCTTTGAGGCTCTCAAACAGTTAGTCGTAGCTGGTAACGTCCTGCTGTACCTACCGGAACCGGAAGGGTCAAACTATAATCCCATGAAGCTGTACCGAtt                                                                                                                    <  1:572444/296‑1 (MQ=255)`  
                                                                                                                                                                                     `cTCGTGGTCTGAACAATCTAGCCTCTAAGATCATGCTGGCTCTATTCCCTATGCAGACTTGGATGCGACTTACTATATCTGAATATGAAGCAAAGCAGTTACTGAGCGACCCAGATGAACTCGCTAAGGTCGATGAGGGCCTCTCGATGGTAGAGCGTATCATCATGAACTACATTGAGTCTAACAGTTACCGCGTGACTCTCTTTGAGGCTCTCAAACAGTTAGTCGTAGCTGGTAACGTCCTGCTGTACCTACCGGAACCGGAAGGGTCAAACTATAATCCCATGAAGCTGTACCGAtt                                                                                                                    >  1:415235/1‑301 (MQ=255)`  
                                                                                                                                                                                                `aaCAATCTAGCCTCTAAGCTCATGCTGGCTCTATTCCCTATGCAGACTTGGATGCGACTTACTATATCTGAATATGAAGCAAAGCAGTTACTGAGCGACCCCGATGAACTCGCTAAGGTCGATGAGGGCCTCTCGATGGTAGAGCGTATCATAATGAACTACATTGAGTCTAACAGTTACCGCGTGACTCTCTTTGAGGCTCTCAAACAGTTAGTCGTAGCTGGTAACGTCCTGCTGTACCTACCGGAACCGGAAGGGTCAAACTATAATCCCATGAAGCTGTACCGATTGTCTTCTTAtg                                                                                                         <  1:422128/301‑1 (MQ=255)`  
                                                                                                                                                                                                `aaCAATCTAGCCTCGAAGCTCATGCTGGCTCTATTCCCTATGCAGACTTGGATGCGACTTACTATATCTGAATATGATGCAAAGCAGTTACTGAGCGCCCCCGATGAACTCTCTAAGGTCGATGAGGGCCTCTCGATGGTAGATCGTATCATCATGAACTACATTGAGTCTAACAGTTACCGCGTGACTCTCTTTGAGGCTCTCAAACAGTTAGTCGTAGCTGGTAACGTCCTGCTGTACCTACCGGAACCGGAAGGGTCAAACTATAATCCCATGAAGCTGTACCGATTGTCTTCTTAtg                                                                                                         <  1:801958/301‑1 (MQ=255)`  
                                                                                                                                                                                                           `agatgtgtataagagacagctATTCCCTATGCAGACTTGGATGCGACTTACTATATCTGAATATGAAGCAAAGCAGTTACTGAGCGACCCCGATGAACTCGCTAAGGTCGATGAGGGCCTCTCGATGGTAGAGCGTATCATCATGAACTACATTGAGTCTAACAGTTACCGCGTGACTCTCTTTGAGGCTCTCAAACAGTTAGTCGTAGCTGGTAACGTCCTGCTGTACCTACCGGAACCGGAAGGGTCAAACTATAATCCCATGAAGCTGTACCGATTGTCTTCTTATGTGGTCCAACga                                                                                              <  1:228565/282‑1 (MQ=255)`  
                                                                                                                                                                                                            `tctAAGCTCATGCTGGCTCTATTCCCTATGCAGACTTGGATGCGACTTACTATATCTGAATATGAAGCAAAGCAGTTACTGAGCGACCCCGATGGACTCGCTAAGGTCGATGAGGGCCTCTCGATGGTAGAGCGTATCATCATGAACTACATTGAGTCTAACAGTTACCGCGTGACTCTCTTTGAGGCTCTCAAACAGTTAGTCGTAGCTGGTAACGTCCTGCTGTACCTACCGGAACCGGCAGGGTCAAACTATAATCCCATGAAGCTGTACCGATTGTCTTCTTCTGTGGTCCacagac                                                                                             >  1:619908/1‑296 (MQ=255)`  
                                                                                                                                                                                                                      `tGCTGGCTCTATTCCCTATGCAGACTTGGATGCGACTTACTATATCTGAATATGAAGCAAAGCAGTTACTGAGCGACCCCGATGAACTCGCTAAGGTCGATGAGGGCCTCTCGATGGTAGAGCGTATCATCATGAACTACATTGAGTCTAACAGTTACCGCGTGACTCTCTTTGAGGCTCTCAAACAGTTAGTCGTAGCTGGTAACGTCCTGCTGTACCTACCGGAACCGGAAGGGTCAAACTATAATCCCATGAAGCTGTACCGATTGTCTTCTTATGTGGTCCAACGAGACGCATTCgg                                                                                   >  1:495330/1‑301 (MQ=255)`  
                                                                                                                                                                                                                          `ggCTCTATTCCCTATGCAGACTTGGATGCGACTTACTATATCTGAATATGAAGCAAAGCAGTTACTGAGCGACCCCGATGAACTCGCTAAGGTCGATGAGGGCCTCTCGATGGTAGAGCGTATCATCATGAACTACATTGAGTCTAACAGTTACCGCGTGACTCTCTTTGAGGCTCTCAAACAGTTAGTCGTAGCTGGTAACGTCCTGCTGTACCTACCGGAACCGGAAGGGTCAAACTATAATCCCATGAAGCTGTACCGATTGTCTTCTTATGTGGTCCAACGAGACGCATTCGGCAAc                                                                               >  1:565018/1‑301 (MQ=255)`  
                                                                                                                                                                                                                          `ggCTCTATTCCCTATGCAGACTTGGATGCGACTTACTATATCTGAATATGAAGCAAAGCAGTTACTGAGCGACCCCGATGAACTCGCTAAGGTCGATGAGGGCCTCTCGATGGTAGAGCGTATCATCATGAACTACATTGAGTCTAACAGTTACCGCGTGACTCTCTTTGAGGCTCTCAAACAGTTAGTCGTAGCTGGTAACGTCCTGCTGTACCTACCGGAACCGGAAGGGTCAAACTATAAACCCATGAAGCTGTACCGATTGTCTTCTTATGTGGTCCAACGAGACGCATTCGGCAAc                                                                               >  1:722585/1‑301 (MQ=255)`  
                                                                                                                                                                                                                            `ctctATTCCCTATGCAGACTTGGATGCGACTTACTATATCTGAATATGAAGCAAAGCAGTTACTGAGCGACCCCGATGAACTCGCTAAGGTCGATGAGGGCCTCTCGATGGTAGAGCGTATCATCATGAACTACATTGAGTCTAACAGTTACCGCGTGACTCTCTTTGAGGCTCTCAAACAGTTAGTCGTAGCTGGTAACGTCCTGCTGTACCTACCGGAACCGGAAGGGTCAAACTATAATCCCATGAAGCTGTACCGATTGTCTTCTTATGTGGTCCAACGAGACGCATTCGGCAACGt                                                                             <  1:316260/301‑1 (MQ=255)`  
                                                                                                                                                                                                                                 `agcccTATGCAGACTTGGATGCGACTTACTATATCTGAATATGAAGCAAAGCAGTTACTGAGCGACCCCGATGAACTCGCTAAGGTCGATGAGGGCCTCTCGATGGTAGAGCGTATCATCATGAACTACATTGAGTCTAACAGTTACCGCGTGACTCTCTTTGAGGCTCTCAAACAGTTAGTCGTAGCTGGTAACGTCCTGCTGTACCTACCGGAACCGGAAGGGTCAAACTATAATCCCATGAAGCTGTACCGATTGTCTTCTTATGTGGTCCAACGAGACGCATTCGGCAACGTTATGc                                                                        <  1:250509/299‑1 (MQ=255)`  
                                                                                                                                                                                                                                   `cccTATGCAGACTTGGATGCGACTTACTATATCTGAATATGAAGCAAAGCAGTTACTGAGCGACCCCGAGGAACTCGCTAAGGTCGATGAGGGCCTCTCGATGGTAGAGCGTAGCATCATGAACTACATTGAGTCTAACAGTTACCGCGTGACTCTCTTTGAGGCTCTCAAACAGTTAGTCGTAGCTGGTAACGTCCTGCTGTACCTACCGGAACCGGAAGGGTCAAACTATAATCCCATGAAGCTGTACCGATTGTCTTCTTATGTGGTCCAACGAGACGCATTCGGCAACGTTCTGCaa                                                                      <  1:578617/301‑1 (MQ=255)`  
                                                                                                                                                                                                                                   `aagagacaggACTTGGATTCGACTTACTATATCTGAATATGAAGCAAAGCAGTTACTGAGCGACCCCGATGAACTCGCTAAGGTCGATGAGGGCCTCTCGATGGTAGAGCGTATCATCATGAACTACATTGAGTCTAACAGTTACCGCGTGACTCTCTTTGAGGCTCTCAAACAGTTAGTCGTAGCTGGTAACGTCCTGCTGTACCTACCGGAACCGGAAGGGTCAAACTATAATCCCATGAAGCTGTACCGATTGTCTTCTTATGTGGTCCAACGAGACGCATTCGGCAACGTTCTGCaa                                                                      <  1:270441/292‑1 (MQ=255)`  
                                                                                                                                                                                                                                            `gACTTGGATGCGACTTACTATATCTGAATATGAAGCAAAGCAGTTACTGAGCGACCCCGATGAACTCGCTAAGGTCGATGAGGGCCTCTCGATGGTAGAGCGTATCATCATGAACTACATTGAGTCTAACAGTTACCGCGTGACTCTCTTTGAGGCTCTCAAACAGTTAGTCGTAGCTGGTAACGTCCTGCTGTACCTACCGGAACCGGAAGGGTCAAACTATAATCCCATGAAGCTGTACCGATTGTCTTCTTATGTGGTCCAACGAGACGCATTCGGCAACGTTCTGCAAATGGGGAcg                                                             >  1:391953/1‑300 (MQ=255)`  
                                                                                                                                                                                                                                                 `ggATGCGACTTACTATATCTGAATATGAAGCAAAGCAGTTACTGAGCGACCCCGATGAACTCACTAAGGTCGATGAGGGCCTCTCGATGGTAGAGCGTATCATCATGAACTACATTGAGTCTAACAGTTACCGCGTGACTCTCTTTGAGGCTCTCAAACAGTTAGTCGTAGCTGGTAACGTCCTGCTGTACCTACCGGAACCGGACGGGTCAAACTATAATCCCATGAAGCTGTACCGATTGTCTTCTTATGTGGTCCAACGAGACGCATTCGGCAACGTTCTGCAAATGGTGACTCGTgt                                                        >  1:557367/1‑300 (MQ=255)`  
                                                                                                                                                                                                                                                   `aTGCGACTTACTATATCTGAATATGAAGCAAAGCAGTTACTGAGCGACCCCGATGAACTCGCTAAGGTCGATGAGGGCCTCTCGATGGTAGAGCGTATCATCATGAACTACATTGAGTCTAACAGTTACCGCGTGACTCTCTTTGAGGCTCTCAAACAGTTAGTCGTAGCTGGTAACGTCCTGCTGTACCTACCGGAACCGGAAGGGTCAAACTATAATCCCATGAAGCTGTACCGATTGTCTTCTTATGTGGTCCAACGAGACGCATTCGGCAACGTTCTGCAAATGGTGACTCGTGAcc                                                      <  1:698680/301‑1 (MQ=255)`  
                                                                                                                                                                                                                                                        `aCTTACTATATCTGAATATGAAGCAAAGCAGTTACTGAGCGACCCCGATGAACTCGCTAAGGTCGATGAGGGCCTCTCGATGGTAGAGCGTATCATCATGAACTACATTGAGTCTAACAGTTACCGCGTGACTCTCTTTGAGGCTCTCAAACAGTTAGTCGTAGCTGGTAACGTCCTGCTGTACCTACCGGAACCGGAAGGGTCAAACTATAATCCCATGAAGCTGTACCGATTGTCTTCTTATGTGGTCCAACGAGACGCATTCGGCAACGTTCTGCAAATGGTGACTCGTGACCAGATa                                                 >  1:137140/1‑301 (MQ=255)`  
                                                                                                                                                                                                                                                           `tACTATATCTGAATATGAAGCAAAGCAGTTACTGAGCGACCCCGATGAACTCGCTAAGGTCGATGAGGGCCTCTCGATGGTAGAGCGTATCATCATGAACTACATTGAGTCTAACAGTTACCTCGTGACTCTCTTTGCGGCTCTCAAACTGTTAGTCGTAGCTGGTAACGTCCTGCTGTACCTACCGGAACCGGAAGGGTCAAACTATAATCCCATGAAGCTGTCTCGATTGTCTTCTTATGTGGTCCAACGTGACGCATTAGGCAACGTTCTGCAAATGGTGACGCGTGACCAGATAGCt                                              >  1:473439/1‑301 (MQ=255)`  
                                                                                                                                                                                                                                                           `tACTATATCTGAATATGAAGCAAAGCAGTTACTGAGCGACCCCGATGAACTCGCTAAGGTCGATGAGGGCCTCTCGATGGTAGAGCGTATCATCATGAACTACATTGAGTCTAACAGTTACCGCGTGACTCTCTTTGAGGCTCTCAAACAGTTAGTCGTAGCTGGTAACGTCCTGCTGTACCTACCGGAACCGGAAGGGTCAAACTATAATCCCATGAAGCTGTACCGATTGTCTTCTTATGTGGTCCAACGAGACGCATTCGGCAACGTTCTGCAAATGGTGACTCGTGACCAGATAGCt                                              >  1:510506/1‑301 (MQ=255)`  
                                                                                                                                                                                                                                                           `tACTATATCTGAATATGAAGCAAAGCAGTTACTGAGCGACCCCGATGAACTCGCTAAGGTCGATGAGGGCCTCTCGATGGTAGAGCGTATCATCATGAACTACATTGAGTCTAACAGTTACCGCGTGACTCTCTTTGAGGCTCTCAAACAGTTAGTCGTAGCTGGTAACGTCCTGCTGTAACTACCGGAACCGGAAGGGTCAAACTATAATCCCATGAAGCTGTACCGATTGTCTTCTTATGTGGTCCAACGAGACGCATTCGGCAACGTTCTGCAAATGGTGACTCGTGACCAGATAGCt                                              >  1:540833/1‑301 (MQ=255)`  
                                                                                                                                                                                                                                                             `cTATATCTGAATATGAAGCAAAGCAGTTACTGAGCGACCCCGATGAACTCGCTAAGGTCGATGAGGGCCTCTCGATGGTAGAGCGTATCATCATGAACTACATTGAGTCTAACAGTTACCGCGTGACTCTCTTTGAGGCTCTCAAACAGTTAGTCGTAGCTGGTAACGTCCTGCTGTACCTACCGGAACCGGAAGGGTCAAACTATAATCCCATGAAGCTGTACCGATTGTCTTCTTATGTGGTCCAACGAGACGCATTCGGCAACGTTCTGCAAATGGTGACTCGTGACCAGATAGcctg                                            >  1:627699/1‑298 (MQ=255)`  
                                                                                                                                                                                                                                                                 `atCTGAATATGAAGCAAAGCAGTTACTGAGCGACCCCGATGAACTCGCTAAGGTCGATGAGGGCCTCTCGATGGTAGAGCGTATCATCATTAACTACATTGAGTCTAACAGTTACCGCGTTACTCTCTTTGAGGCTCTCAAACAGTTAGTCGTAGCTGGTAACGTCCTGCTGTACCTACCGGAACCGGAAGGGTCAAACTATAATCCCATGAAGCTGTACCGATTGTCTTCTTATGTGGTCCAACGAGACGCATTCGGCAACGTTCTGCAAATGGTGACTCGTGACCAGATAGCTTTTGGt                                        <  1:693015/301‑1 (MQ=255)`  
                                                                                                                                                                                                                                                                 `atCTGAATATGAAGCAAAGCAGTTACTGAGCGACCCCGATGAACTCGCTAAGGTCGATGAGGGCCTCTCGATGGTAGAGCGTATCATCATGAACTACATTGAGTCTAACAGTTACCGCGTGACTCTCTTTGAGGCTCTCAAACAGTTAGTCGTAGCTGGTAACGTCCTGCTGTACCTACCGGAACCGGAAGGGTCAAACTATAATCCCATGAAGCTGTACCGATTGTCTTCTTATGTGGTCCAACGAGACGCATTCGGCAACGTTCTGCAAATGGTGACTCGTGACCAGATAGCTTTTGGt                                        >  1:13065/1‑301 (MQ=255)`  
                                                                                                                                                                                                                                                                  `tCTGAATATGAAGCAAAGCAGTTACTGAGCGACCCCGATGAACTCGCTAAGGTCGATGAGGGCCTCTCGATGGTAGAGCGTATCATCATGAACTACATTGAGTCTAACAGTTACCGCGTGACTCTCTTTGAGGCTCTCAAACAGTTAGTCGTAGCTGGTAACGTCCTGCTGTACCTACCGGAACCGGAAGGGTCAAACTATAATCCCATGAAGCTGTACCGATTGTCTTCTTATGTGGTCCAACGAGACGCATTCGGCAACGTTCTGCAAATGGTGACTCGTGACCAGATAGCTTTTGGTg                                       >  1:263491/1‑301 (MQ=255)`  
                                                                                                                                                                                                                                                                     `gAATATGAAGCAAAGCAGTTACTGAGCGACCCCGATGAACTCGCTAAGGTCGATGAGGGCCTCTCGATGGTAGAGCGTATCATCATGAACTACATTGAGTCTAACAGTTACCGCGTGACTCTCTTTGAGGCTATCAAACAGTTAGTCGTAGCTGGTAAAGTCCTGCTGTACCTACCGGAACCGGAAGGGTCAAACTATAATCCCATGAAGCTGTACCGCTTGTCTTCTTATGTGGTCCAACGAGACGCATTCGGTCACGTTCTGCAAATGGTGACTCGTGACCAGATAGCTTTTGGTGcct                                    >  1:92498/1‑299 (MQ=255)`  
                                                                                                                                                                                                                                                                              `gcaaagcaGTTACTGAGCGACCCCGATGAACTCGCTAAGGTCGATGAGGGCCTCTCGATGGTAGAGCGTATCATCATGAACTACATTGAGTCTAACAGTTACCGCGTGACTCTCTTTGAGGCTCTCAAACAGTTAGTCGTAGCTGGTAACGTCCTGCTGTACCTACCGGAACCGGAAGGGTCAAACTATAATCCCATGAAGCTGTACCGATTGTCTTCTTATGTGGTCCAACGAGATGCATTCGGCAACGTTCTGCAAATGGTGACTCGTGACCAGATAGCtttctgtctcttatacacat                           >  1:612511/1‑284 (MQ=255)`  
                                                                                                                                                                                                                                                                               `caaagcaTTTACTGAGCGACCCCGATGAACTCGCTAAGGTCGATGAGGGCCTCTCGATGGTATAGCGTATCATCATGAACTACATTGAGTCTAACAGTTACCGCGTGACTCTCTTTGAGGCTCTCAAACAGTTAGTCGTAGCTGGTAACGTCCTGCTGTACCTACCGGAACCGGAAGGGTCAAACTATAATCCCATGAAGCTGTACCGATTGTCTTCTTATGTGGTCCAACGAGACGCATTCGGCAACGTTCTGCAAATGGTGACTCGTGACCAGATAGCTTTTGGTGCTCTCCCTGAGGa                          <  1:728273/301‑1 (MQ=255)`  
                                                                                                                                                                                                                                                                               `aaaagcaGTTACTGAGGGACCCCGATGAACTCGCGAAAGGCGATTAGGGCCTATCGATGGTAGAGCTTATCAACATGAACTACATGGAATTTAACAGTTACCGCGTGACTCTCTTTGAGGCTCTCAAACAGTTAGTCGTAGCTGGTAACGTCCTGCTGTACCTACCGAAACCGGAAGGGTCAAACTATAATCCCATGAAGCGGTACCGATTGTCTTCTTAGGTGGTCCAACGAGACGCATTCGGCAACGTTCTGCAAATGGTGACTCGTGACCAGATAGCTTTTGGTGCTCTCCCTGAGGa                          <  1:191209/300‑1 (MQ=255)`  
                                                                                                                                                                                                                                                                               `aaaagcaGTTACTGAGCGACCCCGATGAACTCGCTAAGGTCGATGAGGGCCTCTCGATGGTAGAGCGTATCATCATGAACTACATTGAGTCTAACAGTTACCGCGTGACTCTCTTTGAGGCTCTCAAACAGTTAGTCGTAGCTGGTAACGTCCTGCTGTACCTACCGGAACCGGAAGGGTCAAACTATAATCCCATGAAGCTGTACCGATTGTCTTCTTATGTGGTCCAACGAGACGCATTCGGCAACGTTCTGCAAATGGTGACTCGTGACCAGATAGCTTTTGGTGCTCTCCCTGAGGa                          <  1:59992/300‑1 (MQ=255)`  
                                                                                                                                                                                                                                                                                `agacaggTTACTGAGCGACCCCGATGAACTCGCTAAGGTCGATGAGGGCCTCTCGATGGTAGAGCGTATCATCATGAACTACATTGAGTCTAACAGTTACCGCGTGACTCTCTTTGAGGCTCTCAAACAGTTAGTCGTAGCTGGTAACGTCCTGCTGTACCTACCGGAACCGGAAGGGTCAAACTATAATCCCATGAAGCTGTACCGATTGTCTTCTTATGTGGTCCAACGAGACGCATTCGGCAACGTTCTGCAAATGGTGACTCGTGACCAGATAGCTTTTGGTGCTCTCCCTGAGGAc                         <  1:75600/295‑1 (MQ=255)`  
                                                                                                                                                                                                                                                                                    `caGTTACTGAGCGACCCCGATGAACTCGCTAAGGTCGATGAGGGCCTCTCGATGGTAGAGCGTATCATCGTGAACTACATTGAGTCTACCAGTTACCGCGTTACTCTCTTTGAGGCTCTCAAACAGTTAGTCGTAGCTGGTAACGTCCTGCTGTACCTACCGGAACCGGAAGGGTCAAACTATAATCCCATGAAGCTGTACCGATTGTCTTCTTATGTGGTCCAACGAGACGCATTCGGCAACGTTCTGCAAATGGTGACTCGTGACCAGATAGCTTTTGGTGCTCTCCCTGAGGACATcc                     >  1:350897/1‑301 (MQ=255)`  
                                                                                                                                                                                                                                                                                      `gTTACTGAGCGACCCCGATGAACTCGCTAAGGTCGATGAGGGCCTCTCGATGGTAGAGCGTATCATCATGAACTACATTGAGTCTAACAGTTACCGCGTGACTCTCTTTGAGGCTCTCAAACAGTTAGTCGTAGCTGGTAACGTCCTGCTGTACCTACCGGAACCGGAAGGGTCAAACTATAATCCCATGAAGCTGTACCGATTGTCTTCTTATGTGGTCCAACGAGACGCATTCGGCAACGTTCTGCAAATGGTGACTCGTGACCAGATAGCTTTTGGTGCTCTCCCTGAGGACATCCGt                   >  1:749875/1‑301 (MQ=255)`  
                                                                                                                                                                                                                                                                                        `tACTGAGCGATCCCGATGAACTCGCTAAGGTCGATGAGGGCCTCGCGATGGTAGAGCGTATCATCATGAACTACATTGAGTCTAACAGTTACCGCGTGACTCTCTTTGAGGCTCTCAAACAGTTAGTCGTAGCTGGTAACGTCCTGCTGTACCTACCGGAACCGGAAGGGTCAAACTATAATCCCATGAAGCTGTACCGATTGTCTTCTTATGTGGTCCAACGAGACGCATTCGGCAACGTTCTGCAAATGGTGACTCGTGACCAGATAGCTTTTGGTGCTCTCCCTGAGGACATCCGTaa                 <  1:525292/301‑1 (MQ=255)`  
                                                                                                                                                                                                                                                                                           `tGAGCGACCCCGATGAACTCGCTAAGGTCGATGAGGGCCTCTCGATGGTAGAGCGTATCATCATGAACTACATTGAGTCTAACAGTTACCGCGTGAGTCTCTTGGAGGCTCTCAAACAGTTAGTCGTAGCTGGTAACGTCCTGCTGTACCTACCGGAACCGGAAGGGTCAAACTATAATCCCATGAAGCTGTACCGATTGTCTTCTTATGTGGTCCAACGAGACGCATTCGGCAACGTTCTGCAAATGGTGACTCGTGACCAGATAGCTTTTGGTGCTCTCCCTGAGGACATCCGTAAGGc              <  1:308614/301‑1 (MQ=255)`  
                                                                                                                                                                                                                                                                                           `tGAGCGACCCCGATGAACTCGCTAAGGTCGATGAGGGCCTCTCGATGGTAGAGCGTATCATCATGAACTACATTGAGTCTAACAGTTACCGCGTGACTCTCTTTGAGGCTCTCAAACAGTTAGTCGTAGCTGGTAACGTCCTGCTGTACCTACCGGAACCGGAAGGGTCAAACTATAATCCCATGAAGCTGTACCGATTGTCTTCTTATGTGGTCCAACGAGACGCATTCGGCAACGTTCTGCAAATGGTGACTCGTGACCAGATAGCTTTTGGTGCTCTCCCTGAGGACATCCGTAAGGc              <  1:592769/301‑1 (MQ=255)`  
                                                                                                                                                                                                                                                                                           `ggAGCGACCCCGATGAACTAGCTAAGGTGGATGAGGGCCTCTCGATGGTAGAGCGTATCATCATGAACTACATTGAGTCTAATAGTTACCGCGTGACTCTCTTTGAGGCTCTCAAACAGTTAGTCGTAGCTGGTAACGTCCTGCTGTACCTACCGGAACCGGAAGGGTCAAACTATAATCCCATGAAGCTGTACCGATTGTCTTCTTATGTGGTCCAACGAGACGCATTCGGCAACGTTCTGCAAATGGTGACTCGTGACCAGATAGCTTTTGGTGCTCTCCCTGAGGACATCCGTAAGGc              <  1:407011/300‑1 (MQ=255)`  
                                                                                                                                                                                                                                                                                                 `aCCCCGATGAACTCGCTAAGGTCGATGAGGGCCTCTCGATGGTAGAGCGTATCATCATGAACTACATTGAGTATAACAGTTACCGCGTGACTCTCTTTGAGGCTCTCAAACAGTTAGTCGTAGCTGGTAACGTCCTGCTGTACATACCGGAACCGGAAGGGTCAAACTATAATCCCATGAAGCTGTACCGATTGTCTTCTTATGTGGTCCAACGAGACGCATTCGGCAACGTTCTGCAAATGGTGACTCGTGACCAGATAGCTTTTGGTGCTCTCCCTGAGGACATCCGTAAGGCTGTAGa        >  1:175560/1‑301 (MQ=255)`  

                                                                                                                                                                                                                                                                                                          `|`                                                                                                                                                                                                                                                                                                           
`GAGAAACGAACAGGACTTGCGGAGGATGGCGCAAAGTCTGTCTATGAGCGTTTAAAGAACGACCGTGCTCCCTATGAGACACGCGCTCAGAATTGCGCTCAATATACCATCCCATCATTGTTCCCTAAGGACTCCGATAACGCCTCTACAGATTATCAAACTCCGTGGCAAGCCGTGGGCGCTCGTGGTCTGAACAATCTAGCCTCTAAGCTCATGCTGGCTCTATTCCCTATGCAGACTTGGATGCGACTTACTATATCTGAATATGAAGCAAAGCAGTTACTGAGCGACCCCGATGGACTCGCTAAGGTCGATGAGGGCCTCTCGATGGTAGAGCGTATCATCATGAACTACATTGAGTCTAACAGTTACCGCGTGACTCTCTTTGAGGCTCTCAAACAGTTAGTCGTAGCTGGTAACGTCCTGCTGTACCTACCGGAACCGGAAGGGTCAAACTATAATCCCATGAAGCTGTACCGATTGTCTTCTTATGTGGTCCAACGAGACGCATTCGGCAACGTTCTGCAAATGGTGACTCGTGACCAGATAGCTTTTGGTGCTCTCCCTGAGGACATCCGTAAGGCTGTAGAAGGTCA  >  NC_001604.1/20246‑20841`

  

Alignment Legend

Aligned base mismatch/match (shaded by quality score): `ATCG/ATCG` `< 3 ≤` `ATCG/ATCG` `< 11 ≤` `ATCG/ATCG` `< 25 ≤` `ATCG/ATCG` `< 36 ≤` `ATCG/ATCG` `< 38 ≤` `ATCG/ATCG`

Unaligned base: `atcg`    Masked matching base: `atcg`    Alignment gap: `‑`    Deleted base: `‑`
